# P-415. Evaluating the prevention potential of hospital-onset bacteremia: comparison between qualitative and Quantitative evaluation

**DOI:** 10.1093/ofid/ofae631.616

**Published:** 2025-01-29

**Authors:** yael pri-Paz Basson, Pnina Shitrit, Hadar Mudrik-Zohar

**Affiliations:** Meir Medical Center, Kfar-Saba, Tel Aviv, Israel; Meir Medical Center, Kfar-Saba, Tel Aviv, Israel; Meir Medical Center, Israel, Kadima-Tzoran, HaMerkaz, Israel

## Abstract

**Background:**

Hospital–Onset (HO) bacteremia pose a significant threat to hospitalized patients, leading to increased morbidity and mortality. The risk for bacteremia is influenced by both inherent and external risk factors. Evaluating preventability of these infections is crucial for developing effective quality measures and prevention strategies, however, current approaches lack standardization.

Previous research ^(1, 2)^ has outlined a method for evaluating the preventability of HO bacteremia, incorporating a structured rating system that accounts for both patient-specific and healthcare-related risks.

Our study aims to assess the preventability of HO bacteremia in a real-world setting and compare quantitative assessments with qualitative evaluations.

**Table 1:**
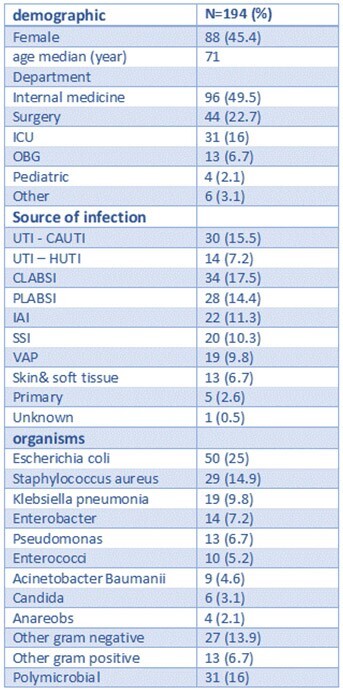
bacteremia events characteristics ICU – intensive care unit; OBG – obstetric and gynecology; UTI – urinary tract infections; CAUTI – catheter associated UTI; HUTI – hospital acquired UTI; CLABSI - Central Line-associated Bloodstream Infection; PLABSI - peripheral line-associated bloodstream infection; IAI – intra abdominal infection; SSI – surgical site infection; VAP - Ventilator-associated Pneumonia

**Methods:**

We conducted a retrospective validation study based on the structured rating system developed by Dantes and Schrank et al. ^(1, 2)^ In this study, we evaluated both intrinsic and extrinsic risk factors contributing to HO bacteremia at Meir Medical Center, Israel. Prevention potential was quantified and compared with qualitative assessments conducted by treating departments and validated by the Infection Control Unit as part of an ongoing hospital wide prevention initiative. ^(3)^

**Table 2:**
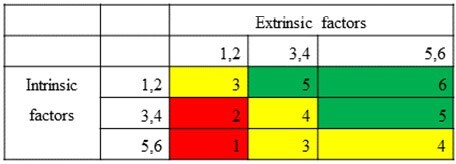
preventability score Preventability score calculated from a one to six (1-6) scale for intrinsic and extrinsic score. 1-2 low prevention potential, 3-4 medium prevention potential and 5-6 indicating high prevention potential.

**Results:**

Analysis of 194 cases from January to December 2022 revealed elevated extrinsic risk factors in the majority of cases, with 51.6% demonstrating significant potential for prevention. There was notable disagreement between the Infection Control Unit and treating departments regarding the perceived preventability of these infections. While the Infection Control Unit deemed 66% of events preventable, departments considered only 20.1% as such. Agreement levels with the preventability rating score were low, with departmental determinations at 0.3 and slightly higher at 0.5 with the Infection Control Unit.

**Table 3:**
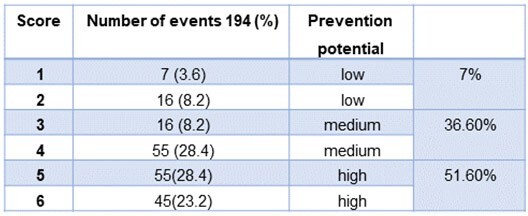
calculated prevention potential

**Conclusion:**

The majority of HO bacteremia have considerable potential for prevention, primarily due to extrinsic risk factors. This underscores the critical need for interventions targeting the understanding and mitigation of these external risks, with the primary objective of reducing HO bacteremia rates and associated morbidity and mortality.

**Figure 1:**
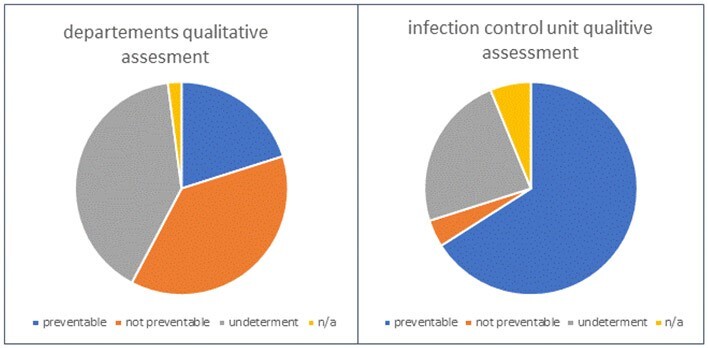
Departments qualitative assessment vs. infection control unit qualitative assessment

**Disclosures:**

**All Authors**: No reported disclosures

